# Tumorigenic bacteria in colorectal cancer: mechanisms and treatments

**DOI:** 10.20892/j.issn.2095-3941.2020.0651

**Published:** 2021-09-30

**Authors:** Sha Li, Jinyi Liu, Xiangjin Zheng, Liwen Ren, Yihui Yang, Wan Li, Weiqi Fu, Jinhua Wang, Guanhua Du

**Affiliations:** 1 The State Key Laboratory of Bioactive Substance and Function of Natural Medicines, Beijing 100050, China; 2 Key Laboratory of Drug Target Research and Drug Screen, Institute of Materia Medica, Chinese Academy of Medical Science and Peking Union Medical College, Beijing 100050, China

**Keywords:** Colorectal cancer, microbiota, tumorigenic mechanism, genotoxicity, cancer pathways, tumor immunity

## Abstract

Colorectal cancer (CRC) is the third most common and the second most fatal cancer. In recent years, more attention has been directed toward the role of gut microbiota in the initiation and development of CRC. Some bacterial species, such as *Fusobacterium nucleatum*, *Escherichia coli*, *Bacteroides fragilis*, *Enterococcus faecalis*, and *Salmonella sp.* have been associated with CRC, based upon sequencing studies in CRC patients and functional studies in cell culture and animal models. These bacteria can cause host DNA damage by genotoxic substances, including colibactin secreted by pks + *Escherichia coli*, *B. fragilis* toxin (BFT) produced by *Bacteroides fragilis*, and typhoid toxin (TT) from *Salmonella*. These bacteria can also indirectly promote CRC by influencing host-signaling pathways, such as E-cadherin/β-catenin, TLR4/MYD88/NF-κB, and SMO/RAS/p38 MAPK. Moreover, some of these bacteria can contribute to CRC progression by helping tumor cells to evade the immune response by suppressing immune cell function, creating a pro-inflammatory environment, or influencing the autophagy process. Treatments with the classical antibacterial drugs, metronidazole or erythromycin, the antibacterial active ingredients, M13@ Ag (electrostatically assembled from inorganic silver nanoparticles and the protein capsid of bacteriophage M13), berberine, and zerumbone, were found to inhibit tumorigenic bacteria to different degrees. In this review, we described progress in elucidating the tumorigenic mechanisms of several CRC-associated bacteria, as well as progress in developing effective antibacterial therapies. Specific bacteria have been shown to be active in the oncogenesis and progression of CRC, and some antibacterial compounds have shown therapeutic potential in bacteria-induced CRC. These bacteria may be useful as biomarkers or therapeutic targets for CRC.

## Introduction

Colorectal cancer (CRC) is the third most common cancer with about 6.1% of all cases and the second most fatal cancer, responsible for 9.2% of all cancer deaths worldwide^1^. There are more than 1 million new cases each year and 600,000 deaths, which involves a huge global economic problem^2^. By 2030, it is predicted that the annual number of new cases will exceed 2.2 million, with 1.1 million deaths^3^. The known environmental risk factors for CRC include smoking, alcoholism, obesity, sedentary lifestyle, diabetes, consumption of red meat, a high fat diet, and insufficient fiber intake^4^. The rising incidence of CRC in developing countries seems to be closely related to changes in lifestyle^4^. Current therapies for CRC include endoscopic and local surgical resection, local ablation treatment of metastases, palliative chemotherapy, targeted therapy, and immunotherapy. Chemotherapy includes monotherapy, based mainly on fluoropyrimidine, as well as multiple drug treatment with oxaliplatin, irinotecan, and capecitabine^5^. Several targeted drugs have also been tested as treatments for CRC, including monoclonal antibodies, cetuximab and panitumumab against the epidermal growth factor receptor, bevacizumab against vascular endothelial growth factor-A, the aflibercept fusion protein, and the small molecule multi-kinase inhibitor regorafenib, all of which target a variety of angiogenesis factors^5^. Although these therapies have doubled the overall survival of patients with advanced disease (up to 3 years), CRC is still associated with poor prognosis and a low long-term survival^6^.

For optimal health, the millions of microorganisms (mainly bacteria) located in the human intestine must flourish in a mutually beneficial balance with the host. The intestine provides a protected nutrient-rich environment for colonizing microorganisms, which in turn help the host by digesting complex carbohydrates, providing non-nutritive essential factors, and occupying niches to resist colonization by pathogens^7^. With increased interest in the gut microbiota and in-depth studies revealing the complex relationships between host and gut microbes, growing results have supported the hypothesis that certain bacterial species are involved in the initiation, development, and response to treatments of many cancers, such as gastric, cervical, and colorectal cancers, especially in those areas that are constantly exposed to microorganisms^8^. The bacterial density in the large intestine is approximately 6-fold greater than in the small intestine, and the risk of cancer in the large intestine is 12-fold higher than that in the small intestine^9^.

To understand how intestinal microbes function in CRC, Tjalsma et al.^9^ developed a now widely recognized model called the “driver-passenger,” system based on a canonical model called the “adenoma-carcinoma sequence,” involving a relatively lengthy functional process. During the process, CRC is thought to be initiated when stem cells at the bottom of the villi crypts of intestinal epithelial cells undergo mutations that make them immortal and able to accumulate additional mutations, such as adenomatous polyposis coli (*APC*), the β-catenin gene (*CTNNB1*), *TP53*, Kirsten rat sarcoma (*KRAS*), and myelocytomatosis oncogene (*MYC*)^10^. In the driver-passenger model, the colonic mucosa of patients with a high risk of CRC are congenitally colonized by bacteria, such as *Bacteroides*, *Enterobacter*, and other bacteria, which can function as drivers. In the adenoma-carcinoma sequence model, “passenger” bacteria can easily be converted into drivers when the process of tumorigenesis is accompanied by rupture and bleeding of tissues, which changes the local microenvironment and microbial selective pressure.

Multi-omics technology is rapidly improving our understanding of the relationships between organisms, including the human body and microbes that live in the human body, and disease susceptibilities. In recent years, rapid progress in bioinformatics of the intestinal microbiome has provided a detailed prediction of some possible drivers of CRC, including their differential distribution in CRC, their potential value as a biomarker or prognostic factor, and the mechanisms involved in the roles they play in CRC.

**Figure 1:**
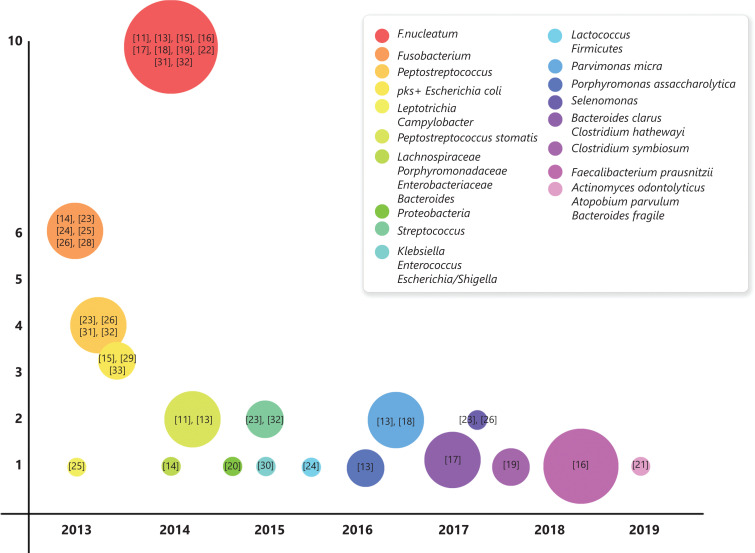
Studies of specific intestinal bacteria of human CRC and normal tissues. The years of the earliest specific bacteria are listed in the abscissa. The total number of studies of a specific bacterium is listed as the ordinate. The circle size represents the relative size of the total number of patients participating in studies involving a specific bacterium. The reference numbers are annotated in circles.

In this review, we have summarized the results of some representative studies^11–35^ (**Figure 1**). Because of the variety of intestinal flora, focusing on certain bacterial species rather than overall changes in the whole microbiome is most important, as the ultimate goal of research is to identify targets for the treatment and prevention of CRC. Species such as *Fusobacterium nucleatum*, *Escherichia coli*, *Bacteroides fragilis*, *Enterococcus faecalis*, and *Salmonella* may be useful as biomarkers for the diagnosis or prognosis of CRC. We also focused on the link between specific bacteria and CRC, the mechanisms involved in their carcinogenic effects, and their potential treatments.

## Specific bacteria associated with CRC

### Fusobacterium nucleatum

*Fusobacterium* is a Gram-negative anaerobic bacterium, which colonizes the human oral cavity, gastrointestinal tract (GIT), and other places. *Fusobacterium* is well-known to be associated with periodontal disease, but has also been isolated from clinical specimens in other diseases, such as appendicitis, brain abscess, osteomyelitis, and pericarditis. Using genomics technology, *Fusobacterium* has been linked to tumorigenesis and the development of CRC, and has become a major focus of research on GIT tumors. In 2012, two groups, from the U.S. and from Canada, reached surprisingly similar conclusions at almost the same time. In the first study^34^, next-generation whole genome sequencing (WGS) was used to characterize the composition of the microbiota in CRC tumors (*n* = 105). The DNA of *Fusobacterium*, mainly *Fusobacterium. nucleatum* (*F. nucleatum*), was enriched in CRC when compared to healthy controls, which was verified by fluorescence *in situ* hybridization (FISH). In another study^35^, shotgun sequencing of DNA of *F. nucleatum* (*n* = 99) was positively correlated with lymph node metastasis in CRC tumors. The *F. nucleatum* strain isolated from CRC tumors was confirmed to promote invasion of human colon epithelial cells (CECs) *in vitro*.

Working within the framework of the driver-passenger model, many investigators now propose that few *F. nucleatum* colonize the intestine under normal conditions. D-galactose-(1-3)-N-acetyl-D-galactosamine (Gal-GalNAc) is overexpressed and *F. nucleatum* binds to it by its outer membrane adhesin protein, Fap2, and accumulates in the intestines of CRC patients^36^. The observation that more than 40% of CRC patients (*n* = 14) had the same *F. nucleatum* strains in saliva as in the intestines^37^ and that oral instillation of *F. nucleatum* was sufficient to aggravate CRC in the *APC ^Min/+^* mouse model, showed a direct oral-digestive tract pathway by which *F. nucleatum* can enter the intestine from the mouth, which differs from the Fap2-dependent hematological pathway^38,39^.

Bacteria in the intestine are separated from the colonic epithelium by a dense mucus layer, which restricts the inflammatory response of the mucosa, thereby allowing tolerance of foreign antigens. Bacteria capable of invading the mucus layer of the colon and forming biofilms can cause chronic mucosal inflammation^40^. The presence of biofilms on the normal mucosa of patients with sporadic CRC has been correlated with tumorigenesis in 89% of the right/proximal CRC and 13% of the left/distal CRC, indicating that biofilms were an important risk factor of CRC^41^. The presence of *F. nucleatum* in CRC tissue has been shown by numerous methods, including rRNA gene amplicon sequencing, DNA sequencing, RNA sequencing, direct quantitative PCR, and FISH. More importantly, this was directly confirmed by the biopsy of CRC patients at different stages,subtypes and races^34,42,43^. Inaddition, more *F. nucleatum* were associated with a shorter survival of CRC patients^38,44^.

### Colibactin and pks + *E. coli*

In 2006, a natural genotoxic compound called colibactin, which can crosslink with eukaryotic DNA and induce double-strand breaks (DSBs)^45,46^, was identified from *Escherichia coli* (*E. coli*) meningitis strain IHE3034a^47^. Colibactin is synthesized by the polyketone compound synthase-nonribosomal peptide synthase assembly line encoded by a 54 kilobase biosynthetic gene cluster called *PKS* (also called *CLB*) island. The colibactin-producing *E. coli* containing the *PKS* island is called pks *+ E. coli*. It is symbiotic^48^ and has been isolated from biological samples of individuals with infectious diseases, such as sepsis^49^ and meningitis in newborns^50^.

Using WGS of human intestinal organoids exposed to *pks + E. coli* strains derived from CRC patients and *E. coli* strains that could not synthesize colibactin, Pleguezuelos-Manzano et al.^51^ found 2 novel mutational characteristics in pks + *E. coli*-related CRC. First, single base substitutions (SBSs) were increased and named as SBS-pks. Major SBSs involved T > N substitutions, including ATA, ATT, and TTT (mutations in the middle base T) and preferentially at the upstream 3 bp adenine. Second, single T mutations in the T homopolymer insertion or deletion were more relevant, which was called ID-pks. Similarly, at the indel site, the adjacent adenine was enriched in the upstream domain. The 2 positively correlated mutational characteristics, SBS-pks and ID-pks, were shown to be related to *APC* mutations that were known to be CRC-driven. Another study revealed that in the presence of colibactin, the intestinal flora was immature and low in diversity. Thus, bacterial gene toxins like colibactin may play an important role at an early stage of life^52^. SBS-pks and ID-pks were detected in 29 of 42 healthy individuals in non-tumor colonic crypts, and data modeling indicated that these features were acquired before 10 years of age. It is therefore possible that individuals with significant PKS mutational characteristics in the early stages of life are at greater risk of developing CRC.

### Bacteroides fragilis

*Bacteroides fragilis* (*B. fragilis*) is an anaerobic bacterium and one of the most common bacilli isolated from biological specimens of patients with diarrhea, peritonitis, intra-abdominal abscess, sepsis, and endogenous purulent infections^53^. *B. fragilis* is classified into nontoxigenic *B. fragilis* (NTBF) and enterotoxigenic *B. fragilis* (ETBF) according to whether the cells secrete the key virulence factor, *B. fragilis* toxin (BFT)^54^. BFT is a zinc-dependent metalloprotease toxin that increases the number of tight junctions in the epithelium by binding CEC receptors^55^. Paradoxically, CECs induced degradation of E-cadherin, which increased permeability, enhanced signaling though the Wnt/β-catenin and NF-κB pathways, and increased BFT binding^55^. Gut colonization by ETBF was significantly correlated with CRC, because an increase in ETBF colonization was detected in approximately 90% of CRC patients^56^, compared to approximately 50% of healthy individuals^56^. Increased ETBF numbers were even found in mucosal biopsies of patients with precancerosis lesions^54^. ETBF colonization was detected in *APC*^Min/+^ mice (adenomatous polyposis locus containing mutant allele for multiple intestinal neoplasia) with inflammatory colitis, which developed into CRC within 4 weeks^53^. The carcinogenic effect of uniformly colonized ETBF was unevenly distributed along the colon axis, usually occurring more frequently at the distal colon, which is similar to human CRC^53^. Contrary to the effect of ETBF, NTBF promoted the development of mucosal immunity and inhibited colitis and CRC by its immunogenic capsule components^57^.

### Other bacteria

A large epidemiological study (*n* = 14,264)^58^ found that the risk of CRC was significantly increased in patients (usually with low malignancy) under 60 years of age who were diagnosed with *Salmonella* infection, especially *Salmonella enteritidis* [SIR 1.54; 95% confidence interval (CI): 1.09–2.10] when compared to the general population. *Peptostreptococcus anaerobius* (*P. anaerobius*) is an anaerobic bacterium selectively enriched in stool samples (*n* = 112) and colon tissue (*n* = 255) of CRC patients^32^. *Enterococcus faecalis* (*E. faecalis*), *Helicobacter pylori*, *Streptococcus bovis*, *Clostridium septicum*, and other intestinal bacteria can also influence the development of CRC^4^.

## Carcinogenic mechanism of specific intestinal bacteria

### Synthesis of genotoxic substances

As a part of their infectious lifecycles, many intestinal bacteria secrete toxins that cause DNA damage to host cells, leading to mutations or deletions in anti-oncogenes or oncogenes. Here, we reviewed the most typical genotoxins, including colibactin, BFT, typhoid toxin (TT), and trans 4-hydroxy-2-nonenal (4HNE). Colibactin, which is produced by pks + *E. coli*, is the most thoroughly investigated genotoxin, and has been shown to cause severe DNA damage and induce CRC in many mouse models^33,40,59,60^. The DNA damage caused by 3 × 10^9^ wild-type pks + *E. coli* cells in the mouse intestinal ring model for 6 h is equivalent to the damage caused by ionizing radiation in 5,000 chest X-ray examination procedures^61^. Studies on the pathogenic mechanism of colibactin over the past decade have been hampered by a lack of structural analyses of the toxin and no direct evidence of its destructiveness to DNA. The complete structural analysis of colibactin is therefore urgently needed to provide information necessary to determine the relationships between *pks* islands and pathogenicity, as well as the mechanism of how pathogenic factors produce genotoxicity at the level of atomic resolution. Colibactin is extremely difficult to isolate because of its low concentration, instability, and its partially resolved biosynthetic pathway involving the heterozygous NRPS-PKS assembly line^47^. The synthesized precolibactin is transported into the periplasm by ClbM, a 12-pass transmembrane transporter. After transport, the prodrug motif is removed *via* deacylation of the pathway-specific serine protease ClbP on the endoplasmic membrane of the bacteria, to generate mature colibactin in the periplasm. The route by which mature colibactin is transported from the periplasm to the target cell is still unknown.

Although efforts to characterize the structure of active colibactin using the structure of precolibactin isolated and identified from the ClbP mutant, pks + *E. coli*, were unsuccessful, several precolibactins containing cyclopropane structures capable of DNA alkylation^59^ were accidentally discovered^46,62–64^. Based on these findings, a hypothesis was proposed that colibactin covalently modified DNA through a cyclopropane moiety. However, this hypothesis could not be tested because of a lack of direct evidence. However, Wilson et al.^65^ recently reported that colibactin synthesized by bacteria colonizing the human body could alkylate DNA to produce DNA adducts and mediate genotoxicity. These results strengthened the role of pks + *E. coli* in the pathogenesis of CRC. Structural representation of the genetic damage induced by colibactin was achieved using innovative liquid high-resolution accurate mass chromatography-resolution mass spectrometry (LC-MS) DNA adductomic analysis. Two putative stereoisomeric DNA adducts were identified in Hela cells infected by pks + *E. coli* and CECs isolated from mono-colonized germ-free mice. New synthetic colibactin mimics were used as standards to determine the structure of the observed DNA adducts, which allowed the investigators to examine how they were formed and provided direct evidence for the presence of adducts *in vivo*, by making structural changes in the colibactin mimics. Xue et al.^66^ recently used an interdisciplinary approach combining total chemical synthesis, metabolomics, and probe-mediated natural product capture (using DNA as a probe, combined with isotopic labeling) and tandem mass spectrometry to deduce the structure of colibactin residues bound to 2 DNA bases. They showed that colibactin was formed by combination of 2 complex biosynthetic intermediates, which produced an almost symmetrical structure consisting of 2 cyclopropane “warheads” in which the rings opened after nucleotide addition. However, they presented no direct evidence regarding structural characterization of the adducts or the biologically relevant DNA reaction environment.

In addition to cyclopropane, an electrophilic unsaturated imine produced by wild-type pks + *E. coli* acted as a DNA disrupting agent and may also be responsible for pks island cytotoxicity. Once ClbP is functionally deactivated, the terminal N-myristoyl-D-Asn side chain continuously converts the intermediate into non-genotoxic pyridone instead of unsaturated imine, abolishing the cytopathic effect of pks islands^62^. The α,β-unsaturated imine derived from dehydration of the intramolecular ring triggered by active colibactin has been shown to enhance the electrophilic reactivity of cyclopropane to adenine residues in DNA^62^.

Colibactin-induced DSBs are probably the result of activation of the host DNA repair mechanism in response to DNA damage, because cultured human cells infected with pks + *E. coli* exhibit interchain cross-links, as well as replication pressure, ATR activation, and FANCD2 recruitment^67^. Deletion of ClbS, a self-protective protein that converts colibactin into harmless compounds *via* its cyclopropane hydrolase activity, and the nucleotide excision repair (NER) protein, UvrB, both show increased toxicity to pks + *E. coli*, inhibiting its growth and highlighting the role of NER in repairing colibactin-induced DNA damage^68^. A recent study has shown that colibactin-induced DSBs were dependent on metal ions with redox activity, which is similar to the activity of the well-known DSB inducer, bleomycin^69^. Li et al.^70^ found that the largest identified precolibactin so far, Precolibactin-969, was hydrolyzed by ClbP to release a water-soluble colibactin containing a macrocyclic skeleton, which was named colibactin-645. It was verified by LC-MS that colibactin-645 was naturally produced by pks + *E. coli* and its degradation was sensitive to trace metals. The recovery was significantly increased when pks + *E. coli* was treated with metal chelating agents like EDTA. In the presence of Cu (II) instead of Fe (II) or Fe (III), colibactin-645 caused DNA fragmentation in a concentration-dependent manner. This process was the result of coupled strand cleavage events resulting in DSBs, rather than the accumulation of unrelated single-strand breaks (SSBs). The macrocyclic skeleton structure of colibactin-645 may be the active center that binds Cu (II) and reduces it.

Although the effect of colibactin on CRC has been clear, there are still many key issues to be resolved by further studies before it can be considered an effective target. For example, given that the genotoxicity of colibactin cannot be exerted through the culture supernatant and lysate of cells infected with pks + *E. coli*, but depends on direct cell contact^47^, how does colibactin enter the host cell nucleus from the bacteria? When considering the specificity of different types of DNA repair pathways for specific DNA damage, knowing which pathways respond to colibactin-induced DNA damage is critical for understanding how it induces susceptibility of CRC. In addition to attacking the host’s DNA, does colibactin contribute to the colonization, persistence, and survival of other bacteria in host tissues? What are the kinetics and relative levels of single adducts compared to the crosslinks between DNA strands caused by alkylation after exposure to pks + *E. coli*? Can we distinguish the precancerous tissue from healthy epithelium by these adducts? Does the failed repair of adduct cause genetic mutations or loss of responsiveness to treatments associated with known CRC subtypes?

*Salmonella* excrete TT, which is structurally and functionally homologous with DNase I. TT, like colibactin, has genotoxicity and can induce SSBs, DSBs, and ATM-dependent DNA damage responses (DDRs)^71^. Following activation of DDR, cells are arrested at the G1 or G2 phase and will eventually undergo senescence or apoptosis if DNA repair fails. However, some of these cells can survive and acquire carcinogenic characteristics including genomic instability^72^. It was confirmed that *Salmonella* activated the PI3K signal pathway, which caused genomic instability in 2-dimensional and 3-dimensional CRC tissue models associated with DNA impairment and failures of cell cycle arrest^73^. The relocation of the Mre11-Rad50-Nbs1 complex is one of the significant features of DDR activation^74^. Investigators have hypothesized that the conserved C-terminal motif of Nbs1 in the Mre11-Rad50-Nbs1 complex interacts with the p110α catalytic subunit of PI3K, leading to the activation of the PI3K signal pathway and genomic instability^71^; however, this hypothesis needs further confirmation.

*E. faecalis* has been shown to cause DNA mutations through production of superoxide compounds and oxygen free radicals, including hydrogen peroxide and hydroxyl radical, which can activate macrophages through a series of redox reactions^74,75^. COX2 expression is upregulated in macrophages, and the non-prostaglandin byproducts, including 4-hydroxy-2-nonenal (4HNE), can invade and integrate with DNA adjacent cells, leading to chromosome instability, aneuploidy, and tetraploidy^76–78^. It has been reported that *B. fragilis* were significantly enriched in CRC tissues with deficient mismatch repair, whereas there were few *B. fragilis* in CRC tissues with proficient mismatch repair^79^. DNA damage can also be caused by *B. fragilis* through the secretion of BFT. BFT is known to activate histone H2AX, the promoter of DNA repair, to initiate rapid repair of DNA damage^80^.

### Changes of signaling pathways in host cells

Several classic cancer-signaling pathways have been shown to participate in the promotion of CRC by intestinal bacteria. For example, the Wnt/β-catenin signaling pathway is activated after binding of *F. nucleatum*a FadA to E-cadherin, resulting in the nuclear transposition of β-catenin and overexpression of Wnt, inflammatory genes, oncogenes like *C-MYC*, and cyclin D1 (*CCND1*). The integrated FadAc complex is comprised of pre-FadA (129 amino acids) and mFadA (111 amino acids) without a signal peptide, and is necessary for the binding reaction between FadA and E-cadherin^81^ in which annexin A1(ANXA1) is a key regulatory factor. ANXA1, also called lipocortin I, is a member of the annexin family of Ca^2+^-dependent phospholipid-binding proteins that are upregulated in sentinel lymph nodes of CRC patients^80^. After being activated, ANXA1 is transported from the cytoplasm to the cell membrane where it is secreted and acts as a ligand for signal transduction through a 7-spanning transmembrane G protein-coupled receptor, formyl peptide receptor 2 (FPR2), also called ALXR in *Homo sapiens*^82^. Studies have showed that *F. nucleatum*-mediated growth stimulation only acted on cancer cells and increased CCND1 expression induced by N-methyl-N’-nitro-N-nitrosoguanidine mutagenesis. Studies investigating the compositional differences between the membranes of cancer cells and non-cancer cells^83^ found that the expression of ANXA1 on the membranes of cancer cells as well as the binding strength between FadA and E-cadherin were significantly higher than on non-cancer cells. Transfection of non-cancer cells with ANXA1 increased their aggressiveness and the binding strength^83^; but the mechanism by which ANXA1 regulates the binding of FadA and E-cadherin remains unknown. Although it is well-known that *F. nucleatum* promotes CRC *via* E-cadherin, it does not stimulate the growth of lung, prostate, and breast cancer cells expressing E-cadherin, or bladder cancer cells without E-cadherin expression. Instead, *F. nucleatum* inhibits the proliferation of these cancer cells as a result of its toxic effects^83^. Even in the presence of E-cadherin, FadA is unable to promote the growth of non-cancer HEK293 cells^84^. This raises several questions for future studies: is the growth stimulating effect of *F. nucleatum* specific for CRC, and if so, how is this specificity achieved? *F. nucleatum* cells, especially those containing lipopolysaccharide (LPS) in the outer membrane^85^, are recognized by Toll-like receptor 4 (TLR4), the main receptor for bacterial LPS. TLR4 activation activates the nuclear factor-κB (NF-κB) pathway through myeloid differentiation primary response gene 88 (MYD88) signaling^86^. NF-κB up-regulates the expression of miR-21, a key promoter of colitis-associated colon cancer^87^, which in turn reduces RAS GTPase RASA1, a member of the RAS-GTPase-activating protein (RAS-GAP) family that binds to and inactivates the oncoprotein, RAS, and inhibits CRC^88^.

Besides DNA damage, ETBF can also affect cancer signaling pathways within host cells. BFT initiates the early and rapid release of CEC mediators, activating Stat3 in immune cells, which in turn induces IL-17 production and subsequent Stat3 activation in CECs^89^. However, Stat3 signaling alone is insufficient for ETBF-mediated tumorigenesis in the distal colon^90^. This is in part because of the synergistic effects between IL-17 and the downstream effector of the activated NF-κB pathway, which stimulate the release of CXC chemokine from CECs, and thereby promote the accumulation of immature CXCR2 + polymorphonuclear bone marrow cells in the lamina propria of the distal colon^90^. BFT also mediates E-cadherin/B-catenin^86^, NF-κB activation^91^, and the p38 mitogen-activated protein kinase (MAPK)^92^ signaling pathway to induce translation of c-myc in CECs and secretion of IL-8, a promotor of epithelial cell proliferation and local angiogenesis in CRC^93^, resulting in sustained cell proliferation^94^. BFT stimulates the expression of MCP-1 (CCL2), which the up-regulates AP-1 protein, recruits neutrophils to destroy mouse colonic villi^95^, and inhibits apoptosis by activating apoptotic protein 2 (cIAP2), which is the downstream target of the p38 MAPK/COX2/prostaglandin E2 (PGE2) pathway^96^. *In vitro* studies have shown that BFT induced polyamine catalyst spermine oxidase (SMO), which triggered reactive oxidative species (ROS) production, DNA damage, and cell proliferation^97^. Both the pro-inflammatory signaling pathways and the apoptotic signaling pathways described above contribute to the ETBF-induced CRC development.

AvrA, a secretion protein expressed by *Salmonella*, is a deubiquitinase that blocks ubiquitinated E3 ligase and promotes cell proliferation and tumorigenesis by activating the Stat3 and Wnt signaling pathways^98^. AvrA stabilizes β-catenin and IκBα by inhibiting ubiquitination, which in turn up-regulates their downstream target genes, *C-MYC* and *CCND1*, increases the proliferation of CECs, reduces apoptosis, and increases the inflammatory response^99^. Feeding heat-killed *E. faecalis* EC-12 reduces the development of intestinal polyposis induced by the β-catenin signaling pathway from the middle to the small intestine in *APC^Min/+^* mice by inhibiting the transcriptional activity of T-cell factor/lymphoid enhancer factor, a transcription factor involved in the mRNA expression of CCND1 in intestinal polyps^100^.

### Inhibition of tumor immunity

In an *APC^Min/+^* mouse model fed with *F. nucleatum*, in which the NF-κB pathway and a variety of pro-inflammatory cytokines including TNF-α, IL-6, IL-8, IL-1β, IL-17F, IL-21, IL-22, and MIP3A were activated^38,101^, myeloid cells including macrophages, dendritic cells, and myeloid-derived suppressor cells were observed in tumors, consistent with RNA-seq data from patients with a high *F. nucleatum* burden^38^. It was confirmed that *F. nucleatum* mediated the proliferation and migration of macrophages/monocytes that promoted CRC development^102,103^. *F. nucleatum* is a compatible intracellular bacterium that can survive and proliferate in macrophages for up to 72 h. It inhibits macrophage apoptosis by activating the PI3K and ERK pathways^103^. In a colorectal tumor *in situ* mouse model, it was observed that *F. nucleatum* and myeloid-derived suppressor cells (MDSCs) were co-localized in CRC tissue sections^104^. Enrichment with *F. nucleatum* caused an increase in MDSCs among infiltrating cells and decreased T cell abundance in tumor tissues^104^. As the first member of Wnt family to trigger the Wnt/β-catenin signaling cascade, Wnt-1 was considered to play an oncogenic role in CRC^105^. A recent study^106^ found that Wnt-1 was down-regulated in CRC patients, and that this process protected intestinal epithelial cells by preventing invasion of pathogenic bacteria and inhibiting inflammation. These protective effects were abolished by AvrA after *Salmonella* colonization^106^. In response to *Salmonella* invasion, pro-inflammatory cytokines IL-8, IL-6, and granulocyte-macrophage colony-stimulating factor (GM-CSF) were significantly up-regulated in host cells^106^.

In addition to creating a pro-inflammatory environment that promotes oncogenesis and development of CRC, *F. nucleatum* can also remodel the tumor microenvironment to evade the anticancer immune response. Fap2, an outer surface protein of *F. nucleatum*, binds to and activates the inhibitory receptors, T cell Ig, ITIM Domain receptor (TIGIT)^42^, and carcinoembryonic antigen-related cell adhesion molecule 1 (CEACAM1)^107^ expressed by T cells and NK cells, which protect *F. nucleatum* and tumor cells from being killed by immune cells. TIGIT is highly expressed in lymphocytes of CRC tissues^108^, while CEACAM1, which is an inhibitory receptor for various immune cell subsets, is expressed on the surface of numerous types of tumor cells and is considered to be a specific biomarker associated with tumor progression, metastasis, and poor prognosis^108^. In mice with azoxymethane-induced CRC, *P. anaerobius* increased colon dysplasia and the total cholesterol level of CRC cells by activating sterol regulatory element-binding protein 2 (SREBF2)^32^. By interacting with Toll-like receptor 2 (TLR2) and TLR4, *P. anaerobius* increased the reactive oxygen species levels, thereby promoting cholesterol synthesis and cell proliferation^32^.

### Autophagy and tumorigenesis

Autophagy, which is the process of homeostasis responsible for the degradation and recycling of proteins and cellular components, has also been closely associated with tumorigenesis in numerous cancers including CRC^109^. High expression of autophagy genes, such as *beclin-1*, *LC3*, *ATG5*, and *ATG6*, is associated with more aggressive CRC phenotypes^109^. Autophagy also helps host cells resist bacterial infection, because it can directly eliminate invading bacteria from cells and degrade them^110^. It was recently reported that the DNA damage repair process caused by pks + *E. coli* was also autophagy-dependent^111^. *In vitro* and *in vivo* experiments have shown that defects in autophagy result in the intracellular accumulation of sequestosome-1(SQSTM1), a receptor targeting ubiquitinated ligand for degradation through autophagy and proteasomal pathways^112^. SQSTM1directly binds to and inhibits nuclear ring finger protein 168 (RNF168), an E3 ligase essential for histone H2A ubiquitination, which marks DSB sites for DNA repair proteins and DNA damage response^111^. This inhibits the recruitment of DNA homologous recombination repair protein, RAD51, which binds to single-stranded DNA (ssDNA) forming helical RAD51-ssDNA nucleoprotein filaments, which are capable of the search and invasion of homologous DNA sequences^113^ to DSB sites of DNA, leading to a dysfunction of DNA damage repair^111^.

Metastasis occurs in approximately 50%–60% of CRC patients and is the leading cause of death^114^. *F. nucleatum* was observed using FISH in liver metastasis of CRC, suggesting that *F. nucleatum* may be involved in the migration of CRC cells to the metastatic sites^115^. It has been shown that *F. nucleatum* activated autophagy-mediated metastasis of CRC *via* caspase activation and recruitment domain 3 (CARD3, also called RIP2), a serine/threonine/tyrosine kinase with carboxyl-terminal CARD, containing 2 LIR motifs that interact directly with LC3 and are conserved in humans and rodents^116^. CARD3-rich cases are associated with transferred gene signature (*P* = 0.0058) (NES = –1.860461, *P* = 0.0058, FDR = 0.055)^117^. Autophagy has a complex role in cancer metastasis because it promotes metastasis by increasing adhesion plaque renewal, neural-free impedance, and metabolic coupling to the tumor matrix, but also inhibits metastasis by reducing the epithelial/mesenchymal transition (EMT), tumor fibrosis, and the Rho GTPase activity^110^; more research is therefore needed to elucidate the mechanism described above. The accumulation of *F. nucleatum* was also found in tumor tissues of CRC patients with recurrence after chemotherapy, and was related to clinicopathological features^117^. Bioinformatics and functional studies have shown that *F. nucleatum* activated autophagy pathways and promoted CRC resistance to chemotherapy by targeting specific microRNAs and innate immune signal transduction through TLR4 and MYD88.

## Advances in drug research for specific intestinal bacteria

Increased knowledge of the functions and mechanisms of specific bacteria in CRC has prompted investigators to consider antimicrobial treatments to achieve better therapeutic outcomes in CRC patients. Bacterial mucus invasion was detected in surgically removed colon specimens of 70% FAP patients who were not treated with antibiotics, but was not detected in patients who were treated with antibiotics for just 24 h before surgery, suggesting that antibiotics effectively inhibited the formation of intestinal biofilms^40^. Using animal experiments, many classic antibiotics have been found to effectively reduce tumor formation by inhibiting intestinal bacteria. Treatment with metronidazole, which mainly targets anaerobic bacteria, disrupted tumor growth in CRC xenograft mice derived from patients carrying *F. nucleatum*, by reducing the burden of *F. nucleatum* and cell proliferation^115^. Cefoxitin has been shown to completely eliminate ETBF colonies in mice, and decrease the levels of IL-17A in the colon^118^. Erythromycin, a macrolide antibiotic, inhibited the transcriptional activity of NF-κB and AP-1 and the expressions of downstream targets, IL-6 and cyclooxygenase 2 (COX-2) in CRC cells^119^. It also reduced the number of proximal intestinal polyps in *APC*^*Min/+*^ mice by nearly 30% and reduced the expressions of IL-6 mRNA and COX-2 in intestinal polyps^119^. The antibiotics mentioned above that have been proven safe for long-term clinical use are therefore very promising chemo-preventive agents for the treatment of CRC patients.

In addition to conventional antibiotics, other substances including silver ions have also been successfully used to inhibit tumorigenic bacteria^104^. M13@Ag is electrostatically assembled from inorganic silver nanoparticles and the protein capsid of bacteriophage M13, which was selected through phage display technology to specifically bind to *F. nucleatum*^104^. This nanomaterial has been widely used to bind to numerous targets including bacteria, making it possible to specifically inhibit *F. nucleatum*, to greatly improve its effectiveness in the treatment of CRC^104^. The *in vivo* targeting ability and antitumor activity of M13@Ag have been reported, especially when combined with immune checkpoint inhibitors or first-line chemotherapy^106^. The effects of M13@Ag may be a result of activation of antigen-presenting cells along with inhibition of MDSCs and Tregs to mediate the reversal of an immunosuppressive tumor microenvironment (TME)^104^.

Some natural products have also been reported to have therapeutic potential. For example, the isoquinoline alkaloid berberine extracted from the traditional Chinese medicine plant, *Coptis chinensis*, has been shown to be nontoxic and to have specific antibacterial activity^120^. Treatment with berberine reversed the imbalance in intestinal microbiota caused by *F. nucleatum* colonization, and was characterized by an increase in *Tenericutes* and *Verrucomicrobia*^120^. Berberine blocked the secretion of mucosal immune factors, IL-21, IL-22, IL-31, and CD40L in mice, and prevented changes in *F. nucleatum*-induced intracellular signaling pathways^120^. In another example, zerumbone, the main component of *Zingiber zerumbet*, has been reported to have antibacterial, antiinflammatory and antitumor activities, and has been shown to reduce ETBF-induced, intestinal inflammation-related CRC by altering the IL-17, β-catenin, Stat3, and NF-κB pathways^121^.

## Conclusion and prospects

**Figure 2:**
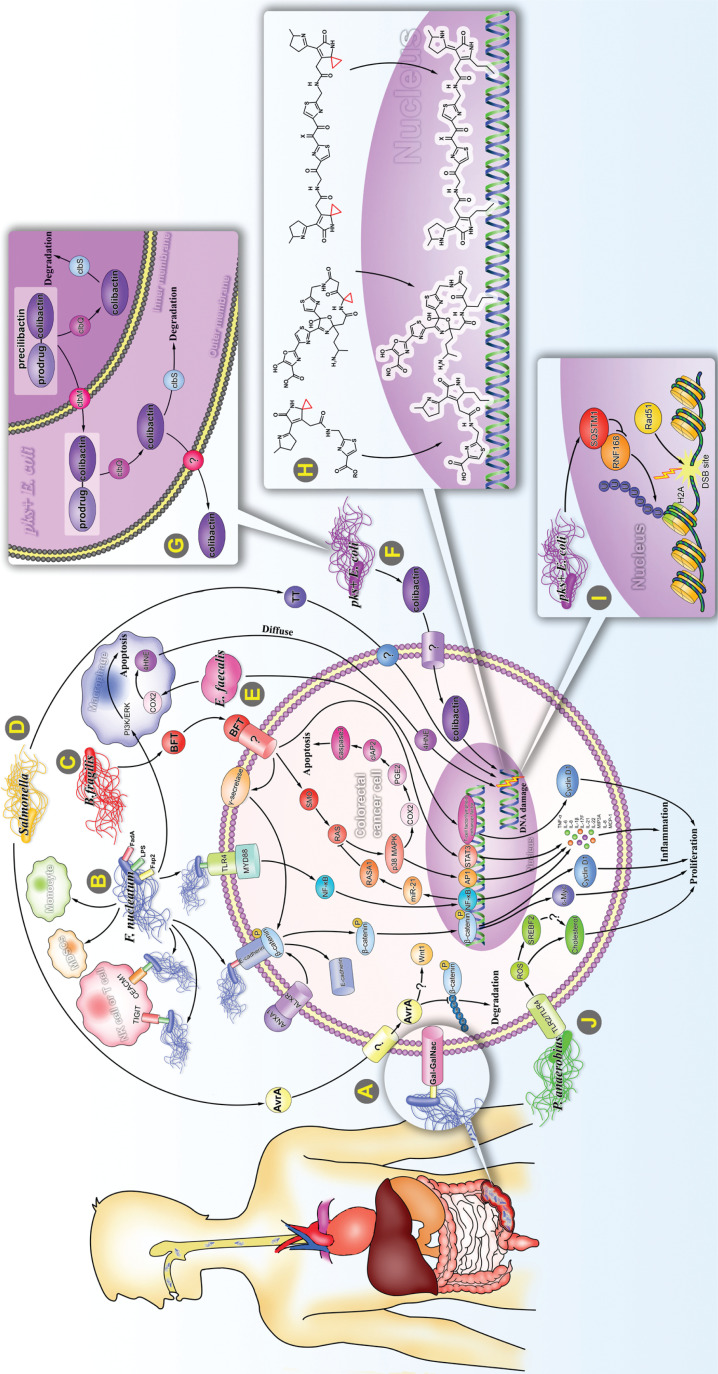
Carcinogenic mechanism of specific intestinal bacteria. (A) Intestinal *F. nucleatum* may be at least partially of oral origin. *F. nucleatum* rarely settles in the intestine, but accumulates in large amounts in the intestine by binding to Gal-GalNAc, which significantly increases when CRC occurs. (B) (1) By binding to E-cadherin, *F. nucleatum* activates Wnt/β-catenin signaling and leads to overexpression of c-Myc and CCND1. This binding process is regulated by ANXA1. (2) *F. nucleatum* binds to TLR4 and activates the NF-κB pathway, thereby enhancing the expression of miR-21, and inhibiting the RAS signal terminator, RASA1. *F. nucleatum* induces expression of multiple pro-inflammatory cytokines by activating the NF-κB pathway. (3) *F. nucleatum* binds to the inhibitory receptors, TIGIT and CEACAM1, of T cells and NK cells and protects *F. nucleatum* and tumor cells from being killed by immune cells. (4) *F. nucleatum* promotes the proliferation and migration of macrophages/monocytes and increases the ratio of MDSCs. (5) *F. nucleatum* inhibits macrophage apoptosis by activating PI3K and ERK pathways. (C) *B. fragilis* mediates Stat3, E-cadherin/B-catenin, NF-κB, and p38 MAPK/AP-1 signaling pathways to induce translation of c-Myc, IL-8, and MCP-1. *B. fragilis* inhibits apoptosis by activating the p38 MAPK/COX2/ PGE2 /cIAP2 pathway triggered by SMO/ROS production. (D) *Salmonella* secretes 2 toxic factors, AvrA and TT. AvrA stabilizes β-catenin and IκBα by inhibiting ubiquitination, which in turn up-regulates their downstream target genes, *c-Myc* and *CCND1*. TT induces DNA single-strand and double-strand breaks (DSBs). (E) *E. faecalis* upregulates expression of COX2 and 4 HNE in macrophages, which could invade and integrate with DNA in adjacent cells. (F) The *pks + E. coli* induces CRC *via* producing a genotoxin called colibactin, which causes severe DNA damage. (G) After processing on the NRPS-PKS assembly line, precolibactin composed of colibactin and prodrug motif is transported into the periplasm by ClbM and deacylated by ClbP in the periplasm to become mature colibactin. It is then transported into the host cell *via* an unknown mechanism. In addition, bacterial cells degrade colibactin through the self-protection protein, ClbS, to protect their own DNA from damage. (H) Several structurally characterized colibactins bind to host DNA by cyclopropane. (I) Intracellularly accumulated SQSTM1 caused by *pks+E. coli* bind to and inhibit the histone H2A ubiquitin enzyme, RNF168, thus inhibiting the recruitment of RAD51 to DSBs sites. (J) By interacting with TLR2 and TLR4, *P. anaerobius* upregulates reactive oxygen species and SREBF2, thereby promoting cholesterol synthesis and cell proliferation.

The tumorigenic mechanisms of intestinal bacteria promoting development of CRC have been summarized (**Figure 2**). With recent advances in genomics, metabolomics, and immunology related to CRC, the discovery of the role of gut microbiota may revolutionize oncotherapy. Although significant progress has been made in recent years, there are still many long-standing issues to be resolved about how intestinal bacteria promote tumorigenesis. First, what is the dominant mechanism among the several possibilities? This is a difficult but critical question to be answered, because it determines the direction of future studies of potential therapeutic targets. There are many factors to be considered, ranging from the physical and chemical conditions of the intestine to the microenvironment of tumor cells. Evidence indicates that diet, nutrition, lifestyle, the environment, the microbiome, and other exogenous factors can have pathogenic roles and can also influence the genome, epigenome, transcriptome, proteome, and metabolome of both tumor cells and non-neoplastic and immune cells^122^. For example, large-scale (*n* = 1,041) clinical research results have indicated that microsatellite instability (MSI) status could affect the tumorigenic mechanism of *F. nucleatum*^123^. In CRC with high MSI status, the patients usually had abundant immunogenic neo-antigens and mounted a stronger antitumor immune response in the tumor microenvironment^123^. In contrast, in CRC patients with low MSI, deficiencies in lymphocyte responses caused by pro-inflammatory components of *F. nucleatum* have emerged as the dominant mechanism^123^. It has been speculated that gene/environment (G × E) interactions could also be important determinants of CRC risk. Genome-wide association studies have shown that up to 50% of CRC inheritability can be explained by common and rare variants included in popular genotyping arrays^124^. In addition to the molecular characteristics of tumors, lifestyle and environmental and genetic factors can also influence tumor cell behavior and affect the clinical outcomes of CRC patients^125^. In the tumor microenvironment, there is a dynamic interactive network that includes neoplastic cells, microorganisms, and immune cells, all of which are affected by the genetic architecture and epidemiological factors including age, diet, nutrition, smoking, alcohol, adiposity, diabetes mellitus, physical exercise, and medications^122^. To better understand how lifestyle and environmental or genetic factors influence tumor cell behavior, the principles of molecular pathological epidemiology (MPE) can be used, which is a relatively new field of epidemiology based on molecular classification of cancers proposed and developed by Ogino et al.^125^. MPE combines the strengths of an interdisciplinary integration of epidemiology, biostatistics, and bioinformatics, and has been used to study breast, lung, prostate, and colon cancers^126^.

Microorganisms, the immune system, and tumor cells interact with each other in a complex manner. Hence, to better understand cancer etiology and its consequences in populations, analyses of the microbiome in various body sites including pathologically altered tissues including tumors should be integrated into MPE (referred to as “microbiology-MPE”)^127^. Microbiology-MPE provides a promising approach for characterizing heterogeneity of the carcinogenic process in relation to microbial composition, and for generating evidence for the role of microorganisms in specific processes of tumor initiation and progression^127^. For example, researchers found that an inflammatory diet rich in red meat and processed meats, refined grains, and sugar was associated with increased risk of *F. nucleatum*-positive CRC^128^. Adherence to a diet based on vegetables, whole grains, fish, fruits, and poultry resulted in a lower incidence of *F. nucleatum*-positive CRC^129^. However, in *F. nucleatum*-negative CRC, no such difference was observed. These findings clearly indicated a role for intestinal microbiota in mediating the association between diet and CRC, supporting the concept that nutritional intervention might be used in specific preventions and treatments of cancers. Although the link between pathogenic inflammation and cancer has become clearer in the past few years, CRC has not been epidemiologically associated with a single microorganism, and there is no strong evidence that alterations in the composition of a single microorganism can significantly affect the incidence and clinical outcome of CRC. This reminds us that more attention should be addressed to changes in several specific bacteria and their possible synergies. For example, pks + *E. coli* and ETBF synergistically promote tumorigenesis in AOM mice in a complementary way. ETBF facilitates the adhesion of pks + *E. coli* and transposition of colibactin to CECs by degrading the mucus, while pks + *E. coli* promoted ETBF-induced IL-17 at an early stage^40^. The low selectivity of antibiotics is an inevitable problem when antibiotic therapy is considered. Some probiotics, such as *Lactobacillus*, *Bifidobacterium* and NTBF, were shown to mitigate DNA damage by promoting the renewal of the epithelium, reducing the accumulation of Th17, regulating the major histocompatibility complex II in dendritic cells, and improving the recruitment and cytotoxicity of natural killer cells and cytotoxic T cells^4^. Treatment with antibiotics, especially broad spectrum antibiotics, will inhibit pathogenic intestinal bacteria but will also inhibit beneficial probiotics, with complex and profound impacts on the entire intestinal flora. Thus, the goal should be to eliminate pathogenic bacteria while simultaneously maintaining the beneficial intestinal microbiota, especially probiotics. It is therefore feasible to use narrow-spectrum biotherapeutics, such as bacteriocin-producing probiotics, to fulfill this requirement^96^.

In addition to screening inhibitors of these pathogenic bacteria, we can also take advantage of the distribution differences between CRC and normal tissues to use attenuated or non-pathogenic facultative anaerobic bacteria as drug delivery systems that can survive and complete delivery, even in an extremely hostile environments including local hypoxia caused by radiotherapy and necrosis after surgery. For example, a non-pathogenic *E. coli* strain encoding a SNP in CD47nb was designed to enhance the activation of infiltrating T cells, induce rapid tumor regression, block metastasis, and improve survival in lymphoma in a mouse model^129^. Although the feasibility of this type of immunotherapy for CRC has been verified only for *Salmonella typhimurium* strains^85^, it is a promising research field, which exploits the use of the enriched intestinal bacteria in CRC for immunotherapy.
